# Supplementary material for: Identification of photoperiod-induced specific miRNAs in the adrenal glands of Sunite sheep (Ovis aries)
Source: Front Vet Sci. 2022 Jul 22;9:888207. doi: 10.3389/fvets.2022.888207 (PMC9354845; doi:10.3389/fvets.2022.888207)
Supplement: Supplementary file 1 [file Data_Sheet_1.ZIP › Supplementary file/miRDeep2 file.zip!/miRDeep2 file/Know_Structure/SPLP42A1/oar-mir-3956.pdf]

miRBase precursor : oar-mir-3956
Total read count : 29
oar-miR-3956-5p read count : 27
oar-miR-3956-3p read count : 2
remaining reads : 0

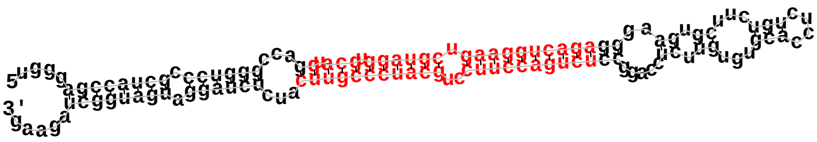

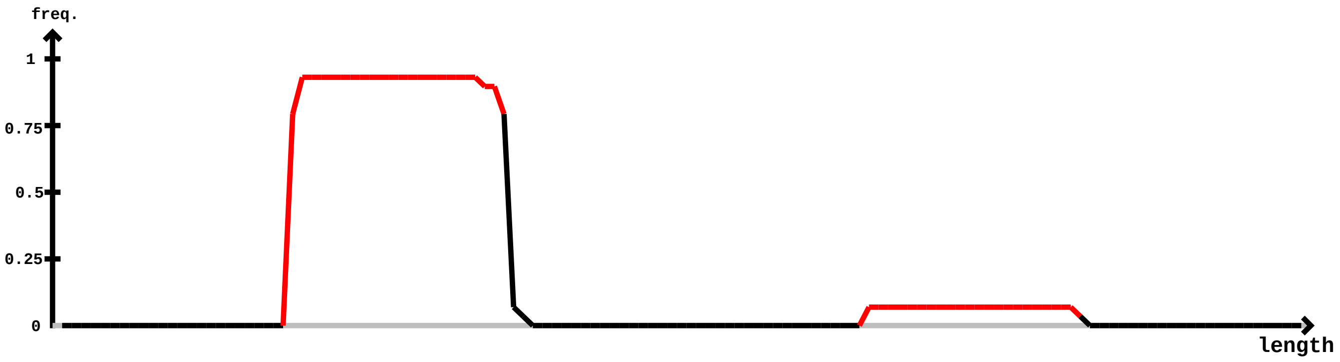

oar-miR-3956-5p

oar-miR-3956-3p

```
5'- ugggagccaucgcccugggccagguacguggaugcugaaggucagagggaagugcuucugucuccacguguguucuccaggccucugaccuuccugcaucccguucaucucuaggaugauggcuagaag   -3'    exp
    ....((((((((.((((((...((.(((.((((((.((((((((((((..((.((...(((....)))...)).)).....))))))))))))..)))))))))))...)))))).)))))))).....    reads      mm
    .......................guacguggaugcugaagguc......................................................................................    1          0
    .......................guacguggaugcugaaggucag....................................................................................    3          0
    .......................guacguggaugcugaaggucaga...................................................................................    17         0
    .......................guacguggaugcugaaggucagag..................................................................................    1          0
    .......................guacguggaugcugaaggucagagg.................................................................................    1          0
    ........................uacguggaugcugaaggucaga...................................................................................    4          0
    ...................................................................................ucugaccuuccugcaucccguu........................    1          0
    ...................................................................................ucugaccuuccugcaucccguuc.......................    1          0
```
